# Prevalence of Metabolic Syndrome and Autoimmune Markers in Adults: A Cross-Sectional Study From Türkiye

**DOI:** 10.1155/ije/5589589

**Published:** 2025-10-30

**Authors:** Fatih Öner Kaya

**Affiliations:** Department of Internal Diseases, Maltepe University Faculty of Medicine, Istanbul, Türkiye

**Keywords:** body mass index, ENA, epidemiology, FANA, metabolic syndrome, obesity, Type 2 diabetes mellitus

## Abstract

**Background:**

Identifying undiagnosed cases of metabolic syndrome (MetS) is important because timely recognition may help reduce the risk of associated complications, including cardiovascular disease (CVD), Type 2 diabetes mellitus (T2DM), stroke, nonalcoholic fatty liver disease (NAFLD), and chronic kidney disease (CKD). This study aims to provide a comprehensive evaluation of MetS and obesity levels among patients attending an outpatient clinic.

**Methods:**

This cross-sectional study was conducted at the outpatient clinics of Istanbul, Maltepe University Hospital, Türkiye, between January 2018 and December 2019, involving adults between the ages of 18 and 70. Demographic and lifestyle information (age, sex, marital status, education, employment, smoking, and comorbidities) was collected through a structured questionnaire. Weight, height, waist circumference, and blood pressure were recorded for all participants. Body mass index (BMI), fasting glucose, HDL cholesterol, triglyceride levels, extractable nuclear antigen (ENA) profiles, and fluorescent antinuclear antibody (FANA) levels were assessed. The Turkish Endocrinology and Metabolism Society's criteria were used to diagnose MetS.

**Results:**

Of the 504 study participants, 55.9% (*n* = 282) were female, with a mean age of 52.2 ± 10.8 years. The overall prevalence of MetS was 32.7% (*n* = 165), with 32.9% (*n* = 93) in females and 32.4% (*n* = 72) in males. Despite similar MetS rates between sexes, lower education and unemployment were associated with higher prevalence. High BMI and a high prevalence of obesity were associated with MetS. Key indicators for MetS diagnosis among participants included weight, height, triglyceride, and HDL levels. The relationship between ENA profiles, FANA levels, and MetS showed significance upon analysis.

**Conclusion:**

In this study, one-third of adults were diagnosed with MetS, with no significant difference in overall prevalence between sexes. Men had lower HDL-C and higher triglycerides, while women showed higher abdominal obesity. In addition, 15.5% of participants were positive for ENA and FANA antibodies despite no clinical autoimmune disease. Socioeconomic disparities, including lower education and unemployment, were also associated with higher MetS prevalence. These findings highlight the importance of integrating both sex-related and socioeconomic factors into targeted screening strategies.

## 1. Introduction

Metabolic syndrome (MetS) is a multifaceted clinical condition defined by several cardiovascular risk factors, such as abdominal obesity, hypertension, low high-density lipoprotein cholesterol (HDL-C), high triglycerides, and insulin resistance [1]. Globally, the prevalence of MetS has risen alongside the epidemic of overweight and obesity, with significant implications for public health. Individuals with MetS are at markedly higher risk for cardiovascular disease (CVD) and Type 2 diabetes mellitus (T2DM) [1, 2]. According to epidemiological data, approximately one-third of adults are affected, highlighting the urgent need for early identification and preventive strategies [3, 4].

Sex-related differences play an important role in the manifestation of MetS. Men are more prone to hypertriglyceridemia and lower HDL-C levels, while women typically exhibit higher rates of abdominal obesity [5]. These differences may reflect variations in hormonal profiles, fat distribution, and lifestyle patterns, and they are crucial for tailoring prevention and management strategies. Understanding the impact of sex on MetS prevalence and its components provides an opportunity for more targeted approaches to risk stratification [3, 4].

Several chronic diseases have obesity and MetS as key risk factors. Early diagnosis of MetS allows for prompt interventions that lessen the risk of various chronic diseases, especially cardiovascular issues; this explains the extensive global research on the subject [6–8]. Beyond metabolic risk, growing evidence suggests a close interplay between MetS and autoimmune processes. Chronic low-grade inflammation and adipokine dysregulation, hallmarks of obesity and MetS, may contribute to immune system activation and autoantibody production [9, 10]. Several studies have reported a higher prevalence of MetS among patients with autoimmune diseases such as systemic lupus erythematosus (SLE) and rheumatoid arthritis (RA), thereby increasing their cardiovascular morbidity [11–14]. A meta-analysis by Janani et al. [11] found that the global prevalence of MetS among patients with inflammatory bowel disease, particularly ulcerative colitis, is notable, highlighting chronic inflammation as a key contributing mechanism. Conversely, immune dysregulation itself may predispose individuals to metabolic abnormalities, suggesting a potential bidirectional relationship [10, 15, 16]. However, data on the association between MetS and autoimmune laboratory markers in the general population remain scarce. This gap underscores the importance of investigating markers such as extractable nuclear antigen (ENA) and fluorescent antinuclear antibodies (FANA) in relation to MetS.

This study aimed to determine participants' sociodemographic characteristics and obesity rates, compare MetS and autoimmune markers by sex, and assess sex-based differences in MetS and autoimmune diseases according to various criteria.

## 2. Materials and Methods

This cross-sectional study included 504 adults (282 females and 222 males) who were followed at the outpatient clinics of Maltepe University Hospital, Istanbul, Türkiye, between January 2018 and December 2019. The study received ethical approval from the Clinical Research Ethics Committee (Approval number: 2023/900/24). Written informed consent was obtained from all participants prior to enrollment.

The minimum sample size was calculated using G∗Power software, assuming a MetS prevalence of 36% for women and 29% for men, a 5% margin of error, 80% power, and a 95% confidence level. The required minimum sample size was 454 participants minimum. In our study, we included 504 individuals, considering the missing data.

### 2.1. Participants and Data Collection

Only those aged 18–70 and not receiving treatment (surgical or medical) for obesity or T2DM were included in this study. Each participant gave informed consent. Aged under 18 or over 70 years old, pregnant, postpartum individuals, and those with incomplete data or who did not consent were excluded. Following informed consent, in line with the Declaration of Helsinki, the study commenced.

Structured questionnaires collected data on participants' demographics, including age, sex, marital status, education, employment, smoking habits, and comorbidities. Socioeconomic status was assessed using education level (illiterate, primary, secondary, high school, and university), employment status (employed/unemployed), and marital status (married/single). Weight (kg), height (cm), and waist circumference (WC) (cm) were measured by an expert using a 150 cm nonstretch tape (Seca 201, Hamburg, Germany). Midway between the iliac crest and the lowest rib, while standing in anatomical position, the WC measurement was taken. Following a 10-min rest, seated blood pressure was measured by an automated sphygmomanometer. Blood pressure was measured three times at five-minute intervals in the seated position, using an automated sphygmomanometer (Omron M6 Comfort, Kyoto, Japan). The first measurement was discarded, and the mean of the last two readings was used for analysis. Patients were classified as hypertensive if their blood pressure was 130/85 mmHg or above [17]. Body mass index (BMI) is calculated as weight (kg) divided by the square of height (m). According to the WHO, BMI categories were classified as underweight (< 18.5 kg/m^2^), normal (18.5–24.9 kg/m^2^), overweight (25–29.9 kg/m^2^), and obese (≥ 30 kg/m^2^) [1].

### 2.2. Assessment of Obesity and MetS

Participants were categorized by BMI (kg/m^2^) to determine the prevalence of obesity. The diagnosis of MetS was based on the criteria of the Turkish Society of Endocrinology and Metabolism (TEMD) [17]:

• Presence of diabetes mellitus, impaired glucose tolerance, or insulin resistance, and at least two of the following:
  1. Hypertension: systolic blood pressure ≥ 130 mmHg, diastolic blood pressure ≥ 85 mmHg, or current antihypertensive therapy.
  2. Dyslipidemia: triglyceride level > 150 mg/dL or HDL-C < 40 mg/dL in men and < 50 mg/dL in women.
  3. Abdominal obesity: BMI ≥ 30 kg/m^2^ or WC > 94 cm in men and > 80 cm in women.

### 2.3. Laboratory and Autoimmune Marker Analysis

Biochemical analyses were performed in the central laboratory using a Roche Cobas c702 (Roche Diagnostics, Mannheim, Germany). Blood samples were obtained after an overnight fast of at least 8–10 h. Biochemical parameters included fasting glucose, triglycerides, HDL-C, and other biochemical markers. ENA (15 parameters) and FANA tests were included as part of the study protocol to explore potential associations between MetS and autoimmune markers, and the costs of these assays were covered by the research budget. Positivity is defined as titers ≥ 1 : 160. Autoimmune markers, such as ENA and FANA profiles, were analyzed using standard immunological methods. Positive ENA and FANA profiles were noted and used in statistical analysis. Percentages of positive ENA and FANA results were calculated based on the total study population, and subgroup analyses were performed separately for females and males. Participants with known clinical diagnoses of autoimmune disease were excluded; only laboratory antibody positivity was assessed in this study.

Measurement error was reduced by using the same instruments for all anthropometric measurements and the same laboratory for all blood tests.

### 2.4. Statistical Analysis

Statistical analyses were performed using IBM SPSS (IBM SPSS Statistics, Version 18.0, Armonk, NY; IBM Corp.). Normality of continuous and descriptive data was checked using Kolmogorov–Smirnov and Shapiro–Wilk tests. Mean ± standard deviation (SD) was reported for continuous variables; frequencies and percentages were reported for categorical variables. Independent sample *t*-tests and chi-square tests were used to analyze continuous and categorical data, respectively. To detect possible sex-related variations, we examined sociodemographic data, MetS indicators, and autoimmune markers for the entire group and then for each sex separately. A *p* value below 0.05 was considered statistically significant.

## 3. Results

A total of 504 participants were included in the study, of whom 282 (55.9%) were female and 222 (44.1%) were male. The mean age of the study population was 52.2 ± 10.8 years (range: 18–70). Sociodemographic characteristics of the participants are shown in Table 1.

The overall prevalence of obesity (BMI ≥ 30 kg/m^2^) was 26.6% (*n* = 134), with a higher proportion in women (15.1%, *n* = 76) compared to men (12.3%, *n* = 62) (*p*=0.001). MetS, defined according to TEMD criteria, was present in 32.7% (*n* = 165) of participants, with similar prevalence between women (32.9%, *n* = 93) and men (32.4%, *n* = 72) (Table 2).

Sex-specific comparisons of individual MetS components and autoimmune markers are presented in Table 3. Abdominal obesity was observed in 60.3% of the total sample, impaired fasting glucose in 42.9%, high triglycerides in 40.9%, low HDL-C in 33.1%, and high blood pressure in 35.9%. In terms of autoimmune markers, 15.5% (*n* = 78) of participants were positive for at least one ENA or FANA parameter (44 women and 34 men).

Socioeconomic characteristics of abdominally obese participants are summarized in Table 4. Women in this subgroup were more likely to be married, unemployed, and less educated compared to men (*p* < 0.001 for all).

Smoking and comorbidity data showed that 28.8% of participants reported current smoking, with a higher prevalence in men than in women (34.2% vs. 24.5%, *p* < 0.05). Hypertension (36.9%), Type 2 diabetes (21.8%), and dyslipidemia (27.4%) were the most common comorbidities identified.

A detailed distribution of ENA profiles across 15 parameters is provided in Table 5. A positive autoimmune result was defined as a titer of ≥ 1 : 160. Among ENA-positive individuals (*n* = 78), anti-Ro/SSA and anti-La/SSB were the most frequently observed antibodies. Among the 504 participants, 78 (15.5%) tested positive for at least one autoimmune marker. Of these, 44 (15.6%) were female and 34 (15.3%) were male. None of these participants had a known clinical diagnosis of autoimmune disease; only laboratory antibody positivity was detected.

## 4. Discussion

This study provides descriptive, population-specific estimates of MetS, its components, and autoimmune markers in adults attending outpatient clinics and explores their variation by sex and socioeconomic factors. MetS affected one-third of participants in our study, irrespective of sex. A 27.4% obesity rate in this study mirrors global patterns, presenting a serious public health concern [1, 2, 7]. In addition, 15.5% of participants tested positive for autoimmune markers.

Given the established cardiometabolic burden of MetS, our findings should be interpreted within this broader clinical context. Overall, MetS prevalence was 32.7% and did not differ by sex (females: 32.9%, males: 32.4%; *p*=0.32). Component-level patterns showed sex-specific profiles; men had lower HDL-C and higher triglycerides, whereas women had higher abdominal obesity. Males and females in the United States of America had MetS prevalence of 39.9% and 38.1%, respectively [2]. As per TEKHARF, a study conducted in Türkiye, 9.2 million individuals aged 30 and above have MetS, and its prevalence has risen by 1.3% over the last 12 years with age [3, 4]. The 32.7% MetS prevalence observed in this study is comparable to rates (20%–40%) reported in similar populations, with variations attributed to differing diagnostic criteria [1, 3, 4, 18]. In contrast to studies showing higher MetS prevalence in females [5], the lack of a sex difference in our study may be attributable to the age composition of our sample or cultural influences on health-related behaviors. These findings are consistent with previous reports showing that women tend to present with higher rates of central obesity, while men more frequently exhibit atherogenic dyslipidemia patterns [3, 5, 15].

Socioeconomic disparities were evident in our cohort. Participants with lower educational attainment and those who were unemployed had a higher prevalence of MetS. This finding aligns with prior evidence showing that socioeconomic disadvantage is strongly associated with cardiometabolic risk factors and MetS [18, 19]. Among abdominally obese participants, women were more likely to be married, less educated, and unemployed, whereas men more frequently reported active employment and higher education levels. These results underscore the importance of considering social determinants of health when addressing the burden of MetS.

In our cohort, 15.5% of adults had positive ENA and FANA profiles, and this finding was observed almost equally in women and men. Although no participant had a confirmed autoimmune diagnosis, antibody positivity may reflect early immune dysregulation. This study implies MetS may play a substantial role in triggering and worsening autoimmune diseases. Inflammation and immune dysfunction are shared mechanisms critically involved in these processes. The impact of MetS requires careful consideration, based on current data, especially regarding conditions such as SLE, seronegative arthritis, autoimmune hepatitis, psoriatic arthritis (PsA), inflammatory bowel diseases, and autoimmune thyroiditis [10, 12, 14]. Proinflammatory cytokines from adipose tissue, especially visceral fat, may play a role in autoimmune processes [10]. Autoimmunity has historically been linked to MetS-related conditions, including obesity, hypertension, and abnormal lipid and glucose metabolism [6, 12, 16]. MetS and RA have overlapping characteristics: high levels of free radicals, insufficiency of antioxidant systems, excessive proinflammatory cytokines, endothelial damage, and unstable atherosclerotic plaques [10, 12, 14]. The percentage of RA patients with MetS is between 14% and 56% [10, 14, 15]. The higher risk of CVD in SLE patients stems from early atherosclerosis and stiffening of blood vessels, features often seen in those with MetS [12, 13, 16]. PsA patients also show a heightened risk of MetS and related CVD [6, 12, 16]. It remains unclear whether MetS promotes the development of autoimmunity through chronic low-grade inflammation and adipokine imbalance, or whether preexisting immune dysregulation predisposes individuals to MetS. Previous studies suggest a bidirectional relationship, where metabolic abnormalities can trigger autoimmune responses, and autoimmunity itself may aggravate metabolic dysfunction [10, 12].

Rare, life-threatening systemic vasculitis is marked by inflammation in the artery or vein walls, resulting in stenosis or thrombosis. MetS is common in patients with systemic vasculitis and contributes to CVD [6, 12, 14, 16]. Cytokines are crucial to idiopathic inflammatory myopathy (IIM) processes. Serum IL-35 levels, for instance, are elevated in IIM patients, acting as a marker for disease activity [12]. Furthermore, the presence of MetS has been linked to the inflammatory pathway, particularly seen in the high incidence of MetS among patients with DM and polymyositis [8, 9, 12, 16]. Previous studies have also reported similar relationships, implying that inflammation due to obesity could intensify autoimmunity in genetically susceptible individuals [15]. In these participants, the absence of identified autoimmune diseases implies possible early or subclinical conditions treatable through lifestyle modifications and weight management. Future research needs to investigate how obesity affects autoimmune markers and disease progression over time.

Abdominal obesity was more frequent in women (62.4%) than men (57.7%), although the difference did not reach statistical significance (*p*=0.087), suggesting a small effect size and/or limited power for this comparison. Similarly, hypertension prevalence was comparable across sexes (31.2% in women vs. 41.8% in men; *p*=0.712), indicating no robust sex difference in this component in our sample. Taken together, these nonsignificant contrasts underscore that sex-related variation in MetS in this setting may be driven more by lipid fractions (HDL-C and triglycerides) than by abdominal obesity or blood pressure.

Untreated high insulin resistance associated with MetS frequently leads to T2DM within a few years, and this is well documented [1, 8]. The proliferation of fat cells, primarily influenced by high insulin levels, can result in inflammation, heightened cytokine levels, increased free radicals, and raised norepinephrine levels [7, 9]. This jeopardizes the health of individuals, escalating preventable risks into permanent ones and heightening the likelihood of morbidity and mortality.

This study has some limitations. The study's limited participation should be viewed. Larger studies may reveal more details on the progression and prevalence of MetS and obesity. Moreover, autoimmune marker rates showed no significant variation between the sexes; the high rate is significant and deserves more study on how metabolic and autoimmune diseases affect each other. This study yields valuable insights but points to areas requiring additional research. Investigating the causal relationships between obesity, MetS components, and autoimmune markers necessitates longitudinal studies. In addition, investigating the genetic and environmental causes of sex differences in metabolic and autoimmune diseases may lead to personalized prevention and treatment.

## 5. Conclusion

In this cross-sectional study, approximately one-third of adults were diagnosed with MetS. While overall prevalence did not differ significantly between sexes, men exhibited lower HDL-C and higher triglyceride levels, whereas women showed higher rates of abdominal obesity. Socioeconomic disparities were also evident, as individuals with lower education and those who were unemployed had higher MetS prevalence. Furthermore, 15.5% of participants were positive for ENA and FANA antibodies despite lacking a clinical autoimmune diagnosis, suggesting a potential subclinical link between MetS and autoimmunity. These findings underscore the need for targeted screening strategies that address both sex-related metabolic differences and socioeconomic risk factors and for future research to clarify the bidirectional relationship between metabolic and autoimmune processes.

## Figures and Tables

**Table 1:** Sociodemographic characteristics of the participants.

Variables		Total *n* = 504
Sex	Female	282 (55.9)
	Male	222 (44.1)

Age^∗^		52.2 ± 10.8

Marital status	Single	123 (24.4)
	Married	381 (75.6)

Working status	Working	135 (26.8)
	Nonworking	369 (73.2)

Smoking	Yes	145 (28.8)
	No	59 (71.2)

Education status	Illiterate	16 (3.1)
	Primary school	109 (21.6)
	Middle school	124 (24.6)
	High school	192 (38.1)
	University	63 (12.6)

Hypertension		181 (35.9)

Dyslipidemia		152 (30.2)

Type 2 diabetes mellitus		97 (19.2)

BMI	Underweight	28 (5.6)
	Normal	124 (24.5)
	Overweight	218 (43.3)
	Obese	134 (26.5)

*Note:* Age is presented as mean ± standard deviation (SD). Categorical variables are presented as frequency (percentage). BMI classification follows the World Health Organization (WHO) criteria: underweight (< 18.5 kg/m^2^), normal (18.5–24.9 kg/m^2^), overweight (25.0–29.9 kg/m^2^), and obese (≥ 30 kg/m^2^).

Abbreviation: BMI = body mass index.

^∗^Age is presented as mean ± standard deviation (SD).

**Table 2:** Distribution of obesity and MetS among participants.

Variables	Female *n* (%)	Male *n* (%)	Total *n* (%)	*p* value^∗^
Obesity (BMI ≥ 30)	74 (14.6)	60 (11.9)	134 (26.5)	0.001
MetS (TEMD criteria)	93 (32.9)	72 (32.4)	165 (32.7)	0.32

*Note:* BMI classification follows the World Health Organization (WHO) criteria: underweight (< 18.5 kg/m^2^), normal (18.5–24.9 kg/m^2^), overweight (25.0–29.9 kg/m^2^), and obese (≥ 30 kg/m^2^).

Abbreviation: BMI = body mass index.

^∗^ *p* < 0.05.

**Table 3:** Individual components of MetS and autoimmune markers by sex.

Variables	Total (*n* = 504)	Female (*n* = 282)	Male (*n* = 222)	*p* value
Abdominal obesity	304 (60.3)	176 (62.4)	128 (57.7)	0.087
Impaired fasting glucose	216 (42.9)	118 (41.8)	98 (44.1)	**< 0.05**
High triglycerides	206 (40.9)	112 (39.7)	94 (42.3)	**< 0.05**
Low HDL-C	167 (33.1)	71 (25.2)	96 (43.2)	**< 0.05**
High blood pressure	181 (35.9)	88 (31.2)	93 (41.8)	0.712
Positive ENA profile	78 (15.5)	44 (15.6)	34 (15.3)	**< 0.05**
Positive FANA	78 (15.5)	44 (15.6)	34 (15.3)	**< 0.05**

*Note:* Values are presented as *n* (%), where *n* represents the frequency, and % represents the percentage within each category. Comparison of differences between females and males and calculation of *p* values were made using chi-square analysis. The bold values, *p* < 0.05 (working status, marital status, and education status), indicate statistically significant differences between sexes.

Abbreviations: ENA = extractable nuclear antigen; FANA = fluorescent antinuclear antibody; HDL = high-density lipoprotein; TEMD = Turkish Society of Endocrinology and Metabolism.

**Table 4:** Socioeconomic status of abdominally obese participants.

		Female *n* = 130	Male *n* = 128	*p* value
Working status	Nonworking	30 (100.0)	43 (33.6)	**< 0.001**
	Working	0 (0.0)	85 (66.4)	

Marital status	Single	0 (0.0)	80 (62.5)	**< 0.001**
	Married	130 (100.0)	48 (37.5)	

Education status	Middle school and lower	97 (74.6)	0 (0.0)	**< 0.001**
	High school	33 (25.4)	92 (71.9)	
	University	0 (0.0)	36 (28.1)	

Hypertension		55 (42.3)	58 (45.3)	0.534

Dyslipidemia		66 (50.8)	57 (44.5)	0.989

Type 2 diabetes mellitus		71 (54.6)	68 (53.1)	0.408

*Note:* Values are presented as *n* (%), where *n* represents the frequency, and % represents the percentage within each category. Comparison of differences between females and males and calculation of *p* values were made using chi-square analysis. The bold values, *p* < 0.05 (working status, marital status, and education status), indicate statistically significant differences between sexes.

**Table 5:** Distribution of positive ENA profiles among participants (*n* = 78).

ENA subtype^∗^	*n* (%)
Anti-Ro/SSA	24 (30.8)
Anti-La/SSB	16 (20.5)
Anti-Sm	12 (15.4)
Anti-RNP	10 (12.8)
Anti-Scl-70	6 (7.7)
Anti-Jo-1	4 (5.1)
Others (e.g., anticentromere, anti-dsDNA)	6 (7.7)

^∗^Positivity defined as titers ≥ 1 : 160.

## Data Availability

The datasets used and/or analyzed during the current study are available from the corresponding author on reasonable request.
